# Role of artificial intelligence in early detection of congenital heart diseases in neonates

**DOI:** 10.3389/fdgth.2023.1345814

**Published:** 2024-01-11

**Authors:** Haris Ejaz, Tarannum Thyyib, Ahmed Ibrahim, Aroob Nishat, Jhancy Malay

**Affiliations:** Department of Pediatrics, Ras Al Khaimah Medical and Health Sciences University, Ras Al Khaimah, United Arab Emirates

**Keywords:** artificial intelligence, screening tools, early diagnosis, neonates, congenital cardiac defects, current challenges

## Abstract

In the domain of healthcare, most importantly pediatric healthcare, the role of artificial intelligence (AI) has significantly impacted the medical field. Congenital heart diseases represent a group of heart diseases that are known to be some of the most critical cardiac conditions present at birth. These heart diseases need a swift diagnosis as well as an intervention to ensure the wellbeing of newborns. Fortunately, with the help of AI, including the highly advanced algorithms, analytics and imaging involved, it provides us with a promising era for neonatal care. This article reviewed published data in PubMed, Science Direct, UpToDate, and Google Scholar between the years 2015–2023. To conclude The use of artificial intelligence in detecting congenital heart diseases has shown great promise in improving the accuracy and efficiency of diagnosis. Several studies have demonstrated the efficacy of AI-based approaches for diagnosing congenital heart diseases, with results indicating that the systems can achieve high levels of sensitivity and specificity. In addition, AI can help reduce the workload of healthcare professionals allowing them to focus on other critical aspects of patient care. Despite the potential benefits of using AI, in addition to detecting congenital heart disease, there are still some challenges to overcome, such as the need for large amounts of high-quality data and the requirement for careful validation of the algorithms. Nevertheless, with ongoing research and development, AI is likely to become an increasingly valuable tool for improving the diagnosis and treatment of congenital heart diseases.

## Introduction

In the domain of healthcare, most importantly pediatric healthcare, the role of artificial intelligence (AI) has significantly impacted the medical field. Congenital heart diseases represent a group of heart diseases that are known to be some of the most critical cardiac conditions present at birth. These heart diseases need a swift diagnosis as well as an intervention to ensure the wellbeing of newborns. Fortunately, with the help of AI, including the highly advanced algorithms, analytics and imaging involved, it provides us with a promising era for neonatal care. This article investigates the groundbreaking capability of simulated intelligence in supporting health care professionals to promptly and precisely recognize congenital heart diseases in neonates, accordingly upgrading their possibilities getting convenient clinical consideration and at last further developing results for these weak newborn children.

## Methodology

The published articles reviewed here were manually collected from multiple databases such as PubMed, ScienceDirect, UpToDate, and Google Scholar for research and reports published between the years 2015–2023. Keywords/combination of keywords used: “Artificial Intelligence”, “Screening tools”, “Early Diagnosis” “Neonates”, “Congenital Cardiac Defects” and “Current challenges”.

## Results

Total twenty-four published full text articles that meet the inclusion criteria were retrieved and analyzed.

## Current techniques to detect CHD in neonates

According to available evidence, neonates with congenital heart defects (CHD) who are identified before birth tend to experience better outcomes compared to those whose condition is detected after they are born. This difference in outcomes can largely be attributed to the frequent delays in diagnosing ductal-dependent CHD, which often occurs when newborns arrive at the emergency department in a state of shock, many days or even weeks after their discharge from the hospital. Until now, no single test, aside from echocardiography, has been demonstrated to be an effective screening technique for identifying CHD in newborns. Recently, pulse oximetry has been suggested as a potential screening method to address the need for reducing the number of infants with serious, ductal-dependent CHD who remain undiagnosed before being sent home from the nursery (1).

### Echocardiography

Echocardiography, a diagnostic imaging technique, is a non-invasive method that employs high-frequency sound waves to create images of the heart (Table 1). This technique is capable of identifying both structural and functional anomalies in the heart. In neonates, echocardiography serves as the primary diagnostic tool for detecting congenital heart defects (CHD). It is used to confirm suspected cases of CHD based on clinical indicators such as cyanosis, heart murmurs, and feeding difficulties (2).

**Table 1:** Clinical implications of echocardiography.

Author	Year	Utility in practice	Future research suggestions
Yoon, S.A. et al.	2020	Echocardiography is crucial in diagnosing CHD in seemingly healthy newborns, improving clinical outcomes for severe cases.	Further research can explore advancements in echocardiographic technology to enhance sensitivity and specificity, especially in early detection.
Zhang, Y.F. et al.	2015	Fetal echocardiography during pregnancy aids in early CHD detection, demonstrating moderate sensitivity and higher specificity.	Future studies can focus on refining fetal echocardiography techniques and addressing variability in examiner experience for more accurate prenatal CHD detection.
Hrusca, A. et al.	2016	Echocardiography is the preferred initial imaging for neonatal CHD, with alternative methods reserved for complex cases.	Research can investigate improvements in echocardiography technology and explore its application in diverse clinical settings.

The utilization of echocardiography to diagnose CHD in seemingly healthy newborns with cardiac murmurs can be advantageous in earlier CHD detection, thereby enhancing the clinical outcomes for newborns with severe CHD (2).

Fetal echocardiography is a specialized form of echocardiography conducted during pregnancy to assess the developing fetus's heart. It can identify CHD as early as 18–22 weeks into gestation. Echocardiography can also uncover asymptomatic instances of CHD, which, if left undetected, can significantly affect long-term outcomes. Prenatal echocardiography for CHD diagnosis demonstrates moderate sensitivity and notably higher specificity (3).

It is recommended for all pregnant women with risk factors for CHD, including a family history of CHD, maternal diabetes, and exposure to certain medications or infections during pregnancy.

The fetal echocardiogram is the primary tool for evaluating and precisely diagnosing fetal cardiovascular abnormalities from the late first trimester until term. The accuracy of prenatal CHD detection via fetal echocardiography can vary significantly, with some of the variability attributed to the experience of the examiner (4). Prenatal CHD detection has the potential to enhance pregnancy outcomes for fetuses with certain types of cardiac defects. Accurate prenatal diagnosis offers valuable clinical advantages concerning infant outcomes.

### Pulse oximetry

Pulse oximetry screening is typically conducted on newborns within 24–48 h after birth as a non-invasive, cost-effective method for identifying critical congenital heart disease (Table 2). While this screening approach has demonstrated effectiveness in detecting critical congenital heart disease in newborns, it cannot reliably detect all types of congenital heart disease. Some forms of congenital heart disease may not lead to significant changes in oxygen saturation, and as a result, they may go unnoticed during pulse oximetry screening (5).

**Table 2:** Clinical implications of pulse oximetry.

Author	Year	Utility in practice	Future research suggestions
Koppel, R. et al.	2003	Pulse oximetry is effective in screening for critical CHD in newborns within 24–48 h after birth.	Future studies can focus on improving the reliability of pulse oximetry for detecting all types of CHD, addressing limitations in sensitivity.

While echocardiography is considered the gold standard for diagnosing congenital heart disease, it typically requires more than 10 min to complete and may not be practical for every newborn, especially in resource-limited areas. In contrast, pulse oximetry provides a convenient and swift alternative, requiring only 2–3 min to analyze the results (5).

### Electrocardiography

ECG (Electrocardiography) is capable of identifying irregular heart rhythms, conduction issues, and other electrical anomalies that might suggest the presence of congenital heart disease (CHD) in neonates (Table 3). In certain instances, ECG results may also offer hints regarding the specific type of heart defect present, although for a reliable diagnosis or exclusion of congenital heart disease, echocardiography remains the preferred method (6). When neonates are suspected of having CHD, an ECG is frequently employed alongside other diagnostic assessments, such as echocardiography, to confirm the diagnosis and evaluate the severity of the defect.

**Table 3:** Clinical utility of ECG.

Author	Year	Utility in practice	Future research suggestions
Narchi, H.	1999	ECG assists in identifying irregular heart rhythms and other anomalies suggestive of CHD in neonates.	Further research can explore advancements in ECG technology for more accurate and rapid CHD diagnosis in neonates.

### Additional imaging

Alternative imaging techniques such as CT (Computed Tomography), CTA (CT Angiography), and cardiac MRI (Magnetic Resonance Imaging) can be employed to identify congenital heart disease in neonates, although they are not the primary imaging methods for this purpose (Table 4). While they have the capacity to detect congenital heart disease in neonates, echocardiography is the preferred initial imaging approach, with these alternative methods reserved for situations where more detailed information is necessary, particularly in complex cases (7).

**Table 4:** Clinical utility of additional imaging.

Author	Year	Utility in practice	Future research suggestions
Hrusca, A. et al.	2016	CT, CTA, and MRI can be employed in complex cases, but caution is needed due to potential risks. Chest x-ray is not reliable for CHD detection.	Future research can focus on minimizing risks associated with CT, CTA, and MRI, and improving their accessibility and applicability in neonatal care.

The utilization of CT and CTA should be approached with caution, considering potential risks to the infant, including radiation exposure. CTA is generally employed where the benefits outweigh the risks, rather than for early detection of congenital heart disease in neonates (7).

On the other hand, cardiac MRI is time consuming, entails sedation or general anesthesia, which can pose risks for neonates.

While a chest x-ray can offer some insights into the heart and lungs of a neonate, it is not a dependable method for detecting congenital heart disease. Congenital heart disease may involve abnormalities in the size, shape, and positioning of heart chambers, blood vessels, and valves, which cannot be accurately identified through a chest x-ray. Consequently, chest x-rays are not routinely used as a screening tool for detecting congenital heart disease in neonates (8).

## Use of artificial intelligence in detection of congenital heart diseases

The utilization of Artificial Intelligence (AI) in various domains within the medical field has been a subject of extensive research and experimentation, aimed at enhancing healthcare and outcomes for a wide range of medical conditions. Over the past decade, there have been notable efforts to employ AI in the context of Congenital Heart Disease (CHD). In this article, we will examine the latest developments and breakthroughs in multiple aspects of machine learning that contribute to the enhancement of CHD screening and risk assessment.

### Prenatal screening

The utilization of machine learning for screening Fetal Congenital Heart Disease (CHD) during gestation, facilitated by the radiation-free and cost-effective nature of echocardiograms, stands as a highly favorable area for implementation (9).

Many studies have been exploring the effectiveness of AI in prenatal ultrasounds and the aim was to aid physician's work. Given that ultrasounds are operator-dependent, inaccuracies resulting from factors like expertise and others significantly impact how the results are interpreted. It has been demonstrated that machine learning can remove these obstacles (10). Algorithms were explored that segments various cardiac ultrasound images such as apical four-chamber view and apical two-chamber view to calculate chambers sizes and stroke volume and various other cardiac indexes to assist the interpreter with labeling anatomical structures, automatized measurements, and provide provisional diagnosis with severity and outcomes. The primary objective is to enhance the efficiency of time management and the precision of diagnostic outcomes. Research has consistently demonstrated improvements in sensitivity and specificity when detecting various Congenital Heart Diseases-(CHD) early in pregnancy. These findings hold the potential to enable earlier diagnosis of fetal cardiac conditions, offering a broader timeframe for implementing interventions that can ultimately reduce mortality rates and lower associated risks (11–13).

The development of algorithms and ongoing clinical trials has highlighted a significant challenge in the deployment of these technologies - the limited availability of data. The data collected from previously diagnosed patients and the inherent anatomical variations among individuals have emerged as critical factors that contribute to elevated rates of false positives and errors in numerous trials (14).

### AI assisted auscultation in CHD detection

Computer-assisted auscultation has become available to assist clinicians with physical examinations to detect congenital heart disease (CHD). In a study comparing physicians with assistance of AI and not shown that, AI-assisted auscultation demonstrated strong sensitivity, good specificity, high accuracy, and excellent agreement with the experts (15).

The electronic stethoscope not only facilitates the recording of heart sounds but also refines them to distinguish between actual and radiating sounds by detecting different frequencies. Moreover, its integration with AI technology enables the digital capture of heart sounds and intelligent identification of pathological murmurs. Clinically hearing subtle heart murmurs can be challenging even for an experienced physician, this implication helps seal that gap, and in turn makes early detection and screening of CHD possible (16).

Artificial intelligence-assisted auscultation (AI-AA) algorithms have been formed by using previous data of abnormal, normal, and subtle murmurs and further classify and form a category to make a system that's able to stratify conditions to suitable diagnosis. These test algorithms performed very well in clinical trials and potentially become used in the near future (17).

As previously discussed in prenatal diagnosis, AI-AA also is data hungry where the more data added the more accurate and less chances of falsely mixing normal with abnormal findings. Several challenges remain to be addressed in the context of AI-assisted auscultation (AI-AA). Foremost, the enhancement of AI algorithms is paramount for achieving high diagnostic accuracy in Congenital Heart Disease (CHD) cases. The clinical collection of heart sound signals often encounters various sources of noise interference, notably the cries of infants and young children (17). This presents challenges for segmentation and classification algorithms, ultimately impacting the accuracy of recognition. Nevertheless, efforts are underway to address these challenges through methods like noise filtering (18).

## Current challenges in implementing AI

Pediatric cardiology, being a cognitively challenging and perceptual subfield involving complex decision-making, makes the use of AI particularly compelling for this medical field. Through simulations and computer programs, we are able to showcase the potential benefits of most AI initiatives; however, progress towards their implementation in actual clinical practice has been more limited (19).

The endless inventory of medical information consisting of diseases, diagnostic methods, biomarkers, and treatment modalities has increased in the past decade, thereby creating an additional need for even more complex decision-making using the vast amount of data available. Machine learning classifiers (MLCs) are one such algorithm that has been used in image-based diagnoses and has already exhibited great aptitude, yet the analysis of diverse and massive electronic health record (EHR) data remains challenging (20).

Some of the challenges faced include the extensive amount of data, high dimensionality, scattered data, and deviations or systematic errors in the medical data itself. These challenges make it difficult to perform accurate pattern recognition and generate predictive clinical models using machine learning techniques (20).

### Vast amount of training data

The quality and quantity of training data have a significant impact on how well the majority of AI algorithms perform. In addition to its expensive nature, the quality of the scanned data differs significantly. The variety in data quality might make it difficult to create a generalized network and poses a significant barrier to the commercialization of AI-based solutions. Other data variations include aberrant anatomical features, MRI machine suppliers, and hospital-specific policies (21).

### Privacy issues

When dealing with medical data, the privacy and security of the patients are the main priorities. These privacy considerations may potentially have a negative effect on the translation of AI models into clinical practice by creating trust and legal issues (21).

### Resource limited settings

There is also the drawback of developing EHRs in resource-limited environments due to concerns with storage capacity as well as a lack of stable, advanced computerized systems to keep such records or data. Even now, many of these developing countries record information in paper formats, which over time may get lost or damaged beyond repair. Transitioning from paper-based records to electronic ones will require massive investments in resources, which most developing countries lack (22).

### Specific limitations

View classification represents another vital step towards building a fully automated echocardiogram interpretation system. Currently, there are additional challenges in creating a view classification model for pediatric echocardiograms compared to adult echocardiograms: multiple variations in anatomy, size, structure, and views (23).

For the development of outcome prediction in the pediatric cardiac ICU, mathematical models that evaluate continuously recorded physiologic parameters, as well as machine learning and AI technologies, are all promising. Since predictive systems are only as successful as the results they anticipate, it is crucial to continue establishing appropriate endpoints as this technology develops. Machine learning might be able to estimate the possibility of certain events, but it won't be able to surpass a physicians' clinical judgment in selecting the best course of treatment given the clinical scenario in order to prevent a clinical decompensation (24). Table 5 provides a concise overview of the advantages and disadvantages of different diagnostic methods, highlighting both traditional and AI-based approaches in diagnosing neonatal congenital heart diseases. Table 6 provides comprehensive discussion of published literature.

**Table 5:** Advantages and disadvantages of diagnostic methods in identifying neonatal congenital heart diseases.

Diagnostic method	Advantages	Disadvantages
Echocardiography	1. Non-invasive method capable of identifying structural and functional anomalies. 2. Primary diagnostic tool for detecting congenital heart defects (CHD) in neonates. 3. Can be used for prenatal diagnosis during pregnancy.	1. Requires skilled personnel for accurate interpretation. 2. Variability in accuracy based on examiner experience. 3. Limited accessibility in resource-limited areas.
Pulse oximetry	1. Non-invasive and cost-effective screening method. 2. Can detect critical congenital heart disease in newborns within 24–48 h.	1. Not reliable for detecting all types of congenital heart disease. 2. May not detect CHD without significant changes in oxygen saturation.
Electrocardiography (ECG)	1. Identifies irregular heart rhythms and electrical anomalies. 2. Can offer hints about the specific type of heart defect.	1. Echocardiography remains the preferred method for reliable diagnosis. 2. Limited in providing a complete diagnosis for congenital heart disease.
Additional imaging	1. Can be employed for more detailed information in complex cases. 2. Computed Tomography (CT) and Magnetic Resonance Imaging (MRI) can detect CHD.	1. Risks associated with radiation exposure in CT and CTA. 2. Cardiac MRI requires sedation or general anesthesia. 3. Limited availability and accessibility in certain medical centers. 4. Not routine for screening in neonates. 5. Chest x-ray is not reliable for CHD detection.
AI in prenatal screening	1. Facilitates screening for Fetal Congenital Heart Disease (CHD) during gestation. 2. Can enhance efficiency and precision in diagnosis.	1. Challenges related to data availability and limited data for AI training. 2. Ongoing clinical trials highlight challenges due to anatomical variations among individuals. 3. Limited availability of data contributes to elevated rates of false positives.
AI-assisted auscultation	1. Demonstrates strong sensitivity, specificity, and accuracy in detecting CHD. 2. Assists in overcoming challenges in clinical auscultation.	1. Challenges related to noise interference during heart sound signals collection. 2. Dependence on high-quality data for accurate classification.
Challenges in implementing AI	1. Offers potential benefits in complex decision-making in pediatric cardiology. 2. Demonstrates aptitude in image-based diagnoses using machine learning classifiers.	1. Faces challenges in analyzing diverse and massive electronic health record (EHR) data. 2. Difficulties in accurate pattern recognition due to extensive medical information. 3. High dimensionality and scattered data present challenges for machine learning techniques.

**Table 6:** Utility of traditional and AI based diagnostic methods in detecting congenital heart diseases in neonates.

Author	Published date	Research methodology	Summary findings	Impact	Disadvantages
Koppel, R., et al. (1)	2003	Review of pulse oximetry effectiveness in CHD detection	Pulse oximetry effective in detecting critical CHD in newborns; reduces undiagnosed cases	Improved outcomes for neonates with early CHD detection	Some CHD types may not be reliably detected; not a comprehensive screening tool
Yoon, S.A., et al. (2)	2020	Review of echocardiography in newborns with asymptomatic cardiac murmurs	Echocardiography is the primary diagnostic tool for CHD in neonates; advantageous in earlier CHD detection	Enhances clinical outcomes for neonates with severe CHD	Time-consuming; may not be practical for all newborns, especially in resource-limited areas
Zhang, Y.F., et al. (3)	2015	Systematic review and meta-analysis of fetal echocardiography for CHD diagnosis	Fetal echocardiography effective in early CHD diagnosis during gestation	Improves pregnancy outcomes for fetuses with cardiac defects	Accuracy varies; examiner experience affects results
Huang, Y., et al. (5)	2022	Evaluation of pulse oximetry screening on newborns	Pulse oximetry as a non-invasive method for identifying critical CHD	Convenient and swift alternative to echocardiography; reduces workload	Cannot reliably detect all types of CHD; may miss some cases
Narchi, H. (6)	1999	Study on neonatal ECG screening for CHD in Down syndrome	ECG identifies irregular heart rhythms; useful alongside echocardiography	Assists in confirming diagnosis and evaluating severity	Limited for reliable CHD diagnosis; echocardiography preferred
Hrusca, A., et al. (7)	2016	Review of alternative imaging techniques for CHD in neonates	CT, CTA, and cardiac MRI can detect CHD; echocardiography preferred	Additional information in complex cases; not primary imaging methods	CT and CTA have potential risks, including radiation exposure; MRI time-consuming and poses risks for neonates
Xu, W., et al. (10)	2020	Study on the use of AI in cardiac auscultation for CHD	AI-assisted auscultation aids in detecting CHD; strong sensitivity and specificity	Improves early detection and screening of CHD	Challenges in noise interference; data-hungry algorithms
Lv, J., et al. (15)	2021	Research on AI-assisted auscultation in CHD detection	AI-AA algorithms classify heart sounds; performs well in clinical trials	Potential future use; aids in stratifying conditions	Challenges in AI algorithm enhancement; noise interference affects accuracy
Van den Eynde, J., et al. (19)	2022	Review on AI in pediatric cardiology	AI initiatives showcase potential benefits; limited progress in clinical implementation	Simulations and programs demonstrate potential benefits	Challenges in vast medical information; diverse and massive EHR data analysis remains challenging
Liang, H., et al. (20)	2019	Evaluation of AI in pediatric disease diagnosis	AI effective in image-based diagnoses; challenges in analyzing diverse EHR data	Promising aptitude in image-based diagnoses	Challenges in data quality, dimensionality, and scattered data
Thomford, N.E., et al. (22)	2020	Study on implementing AI and digital health in resource-limited settings	Lessons learned in implementing AI in congenital heart defects and cardiology	Addresses challenges in resource-limited environments	Concerns with storage capacity; transition from paper-based records to electronic records requires massive investments
Gearhart, A., et al. (23)	2022	Development of an automated view classification model for pediatric echocardiography using AI	AI model for pediatric echocardiography view classification	Aids in automated view classification for pediatric echocardiograms	Challenges in creating a view classification model for pediatric echocardiograms compared to adults
Olive, M.K., et al. (24)	2018	Current monitoring and predictive modeling in the pediatric cardiac intensive care unit	Evaluation of monitoring and predictive modeling in the pediatric cardiac ICU	Promising outcomes in predicting events; limitations in predicting clinical judgment	Challenges in selecting appropriate endpoints; machine learning may not surpass clinical judgment

## Conclusion

The use of artificial intelligence in detecting congenital heart diseases has shown great promise in improving the accuracy and efficiency of diagnosis. By leveraging machine learning algorithms, AI systems can analyze vast amounts of medical data, including images and patient records, to identify potential abnormalities that may indicate a congenital heart condition. Several studies have demonstrated the efficacy of AI-based approaches for diagnosing congenital heart diseases, with results indicating that the systems can achieve high levels of sensitivity and specificity. In addition, AI can help reduce the workload of healthcare professionals allowing them to focus on other critical aspects of patient care. Despite the potential benefits of using AI, in addition to detecting congenital heart disease, there are still some challenges to overcome, such as the need for large amounts of high-quality data and the requirement for careful validation of the algorithms. Nevertheless, with ongoing research and development, AI is likely to become an increasingly valuable tool for improving the diagnosis and treatment of congenital heart diseases.
